# A Novel Splice Variant in the *COL1A1* Gene Leads to Exon 46 Skipping and Osteogenesis Imperfecta

**DOI:** 10.1155/humu/2249583

**Published:** 2026-08-18

**Authors:** Yujun Zhang, Huibing Liu, Ailan Yin, Siping Liu, Wanfei Huang, Yi Zhang, Bei Jia

**Affiliations:** ^1^ Department of Obstetrics and Gynaecology, Nanfang Hospital, Southern Medical University, Guangzhou, China, fimmu.com

**Keywords:** *Col1a1* gene, c-terminal region, minigene splicing assays, osteogenesis imperfecta, splice variants, whole-exome sequencing

## Abstract

**Background:**

Osteogenesis imperfecta (OI) is a clinical and genetic disorder characterised by bone fragility, growth deficiency and skeletal deformity. Ninety per cent of OI cases are attributable to autosomal dominant variants in the *COL1A1* and *COL1A2* genes.

**Methods:**

Candidate variants were identified and verified through trio whole‐exome sequencing (trio‐WES), copy number variation sequencing (CNV‐seq) and Sanger sequencing. Minigene splicing assays were performed in HeLa and HEK293T cells with pcDNA3.1 and pcMINI‐C vectors to investigate the function of the candidate variants. A systematic review of *COL1A1* splicing variants and the corresponding genotype–phenotype spectrum was performed.

**Results:**

Trio‐WES revealed a novel heterozygous variant in the C‐terminal region of the *COL1A1* gene: NM_000088.4:c.3423+5G>A. Sanger sequencing confirmed the variant in both the proband (II‐2) and her foetus (III‐1) who were clinically suspected of having OI. The c.3423+5G>A variant causes complete skipping of Exon 46, as demonstrated by a minigene splicing assay. We retrieved 419 *COL1A1* splicing variants from PubMed, excluded 15 without phenotypic data and 2 linked to Ehlers–Danlos syndrome and stratified the remaining 402 variants into three types on the basis of splice site location: (1) Variants at canonical splicing sites (77.8%, 313/402) mostly cause mild phenotypes, whereas a minority may be severe. (2) Intron variants in other locations, such as splice region variants (17.9%, 72/402), usually cause mild clinical phenotypes, and deep intronic splice variants (0.4%, 2/402) that may result in severe phenotypes. (3) Other variants (3.7%, 15/402), such as exon variants or fragment loss, are extremely rare. We also preliminarily discuss the mechanisms underlying phenotypic variability and the characteristics of C‐terminal variants.

**Conclusions:**

This intron variant in *COL1A1* was classified as likely pathogenic and was confirmed to disrupt *COL1A1* expression. The summary analysis results also revealed a correlation among splicing variants, C‐terminal region variants and disease, suggesting that variant location provides a useful framework for prognosis prediction.

## 1. Background

The *COL1A1* and *COL1A2* genes encode the *α*1 and *α*2 chains of collagen Type I (Col‐I), inherently mimicking the extracellular matrix (ECM) and constituting the predominant protein component in approximately 85%–90% of various tissues, including vasculature, skin, tendon and bone [1]. As a key component in maintaining skeletal structural integrity, Col‐I is the chief organic component of the bone matrix and provides the necessary mechanical strength and toughness for bone tissue. *COL1A1* and *COL1A2* variants may affect the production, synthesis or fixity of collagen fibres in bone [2].

**Figure 1:**
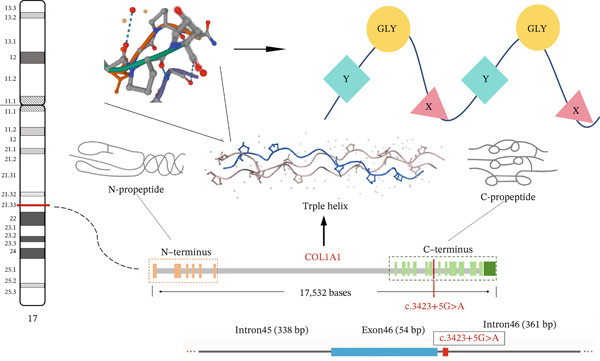
Structure, transcription and expression of the *COL1A1* gene.

The pro Alpha 1 chain, encoded by the *COL1A1* gene, contains a long triple‐helical domain of 1014 amino acid residues in a Gly‐X‐Y sequence and a regulatory C‐propeptide for chain binding. The *COL1A1* gene is located at 17q21.33, spans approximately 17.53 kb and consists of 51 exons encoding a 5914‐bp mRNA that translates into the 1464‐amino‐acid pro‐*α*1 chain. The protein is predominantly localised to the endoplasmic reticulum and the ECM [2, 3]. The transcription and expression processes are detailed in Figure 1. Most previously reported *COL1A1* variants involve amino acid substitutions, insertions or deletions within the triple‐helical domain, leading to abnormal folding of the triple helix [4]. Pathogenic variants in the *COL1A1* gene mainly cause osteogenesis imperfecta (OI, OMIM 166200), also known as brittle bone disorder (OMIM 603828), which is a clinical and genetic disorder characterised by bone fragility, growth deficiency and skeletal deformity. Variants in the *COL1A1* and *COL1A2* genes lead to 90% of OI cases, which is mainly an autosomal dominant disorder characterised by fragile bones, skeletal deformities and growth delay [5]. In addition to OI, variants in the *COL1A1* gene can also cause joint laxity‐type Ehlers–Danlos syndrome or Caffey disease independently, which can cooccur with OI [6, 7]. All of these disorders are autosomal dominant disorders associated with abnormal synthesis of Type I collagen and differ in clinical presentation and prognosis. OI is characterised by skeletal fragility, EDS involves multiple systems, and Caffey disease is characterised by self‐limited infantile periostitis. Differentiation relies on clinical symptoms, imaging studies and genetic testing.

The clinical manifestations of OI are highly variable, ranging from mild bone fragility to perinatal lethality. The Sillence classification [8] categorised the disease into four clinical types based on phenotype and severity. Subsequent types (V–XXIII) have since been added in the OMIM database. Types I–IV are caused by variants in *COL1A1* or *COL1A2* and follow autosomal dominant inheritance; Types V–XVIII and XX–XXIII result from variants in other genes with autosomal recessive inheritance; and Type XIX is X‐linked recessive [9]. In this study, we focus on Types I–IV: Type I, the most common, is usually the mild form, primarily marked by blue sclera and increased bone fragility; Type II is lethal during the perinatal period and is accompanied by severe skeletal deformities; Type III is a gradual deformity that often presents with fracture at birth, severe skeletal deformity and growth delay; and Type IV lies between Types I and III, representing a moderate form, featuring normal sclera and variable bone fragility [8].

To date, a correlation between the *COL1A1* gene and phenotype has been reported. Splice variants in *COL1A1* may cause exon skipping (encoded by Exons 6–49), partial exon deletion or intron retention [4, 10]. Partial structural loss disrupts the triple helical domain, leading to more severe OI phenotypes, whereas exon skipping maintains the reading frame to produce a steady mRNA and generate somewhat functional proteins [4]. We describe a Chinese family with two OI patients harbouring a heterozygous splice variant in the *COL1A1* gene that appeared to have arisen de novo in the proband; this variant was initially classified as a variant of uncertain significance (VUS). We extracted RNA from the proband′s venous blood, performed reverse transcription (RT)–PCR and conducted an in vitro minigene assay to validate that this variant causes severe protein loss and subsequently reclassified it as likely pathogenic. This study expands the genetic and phenotypic landscape of OI and explores its phenotype–genotype correlation.

## 2. Methods

### 2.1. Patients

**Figure 2:**
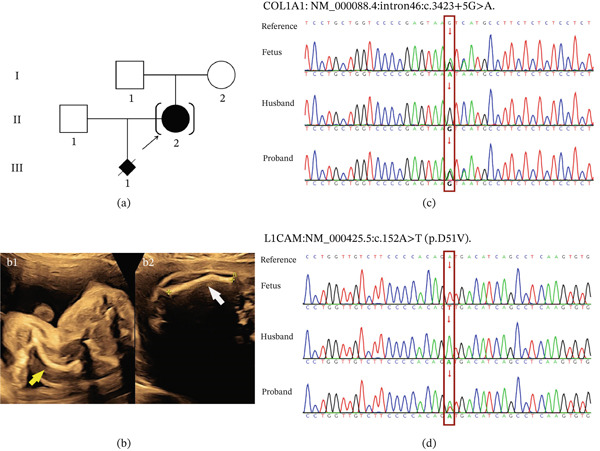
Family pedigree, foetal ultrasound phenotyping and genetic testing results. (a) Family pedigree. (b) Foetal ultrasound phenotyping. Ultrasound revealed hypoplasia of the foetal lower limb bones with limb flexion deformity and right talipes equinovarus. Bilateral humeri, radii and ulnae are visible. Bilateral femora and tibiae are visible with angular deformities of the femora (the left femur b2, yellow arrow) and slight curvature of the right fibula (b1, white arrow). The long‐axis view of the right lower leg shows the plantar aspect of the foot. Foetal femur and tibia measurements and estimated foetal weight are at the 0, 0 and 3.1 percentiles, respectively. (c) Results of Sanger sequencing on NM_000088.4:c.3423+5G>A in the *COL1A1* gene and NM_000425.5:c.152A>T (p. D51V) in the *L1CAM* gene.

The pedigree of the investigated family is shown in Figure 1a. The proband (II‐2), a 25‐year‐old gravida at 28 + 5 weeks of gestation, was included at Nanfang Hospital, Southern Medical University. She was adopted as a family member and presented with bilateral femoral fractures shortly within the first week of life and a tibial fracture during elementary school. Her intellectual, auditory, visual and physical development were normal. Her height was 153 cm, with no obvious skeletal deformities. The sclerae were normally white without a bluish tint. She reported no hearing impairment, and no significant hearing loss was detected on clinical examination. She denied dentinogenesis imperfecta or other dental structural abnormalities but declined further formal audiological and dental evaluations. This was her first pregnancy. Her husband was healthy and had no family history of genetic diseases. Early pregnancy tests, including non‐invasive prenatal testing (NIPT), foetal nuchal translucency (NT) examination and a foetal ultrasound performed at 19 + 5 weeks at another hospital, revealed no significant abnormalities. However, from 22 + 3 weeks of gestation, for the foetus (III‐1), serial ultrasound surveillance demonstrated shortened and slightly curved foetal femurs, with measurements progressively falling from the 6.6th percentile (−1.507 SD) to below the first percentile (−3.767 SD) for gestational age, based on the Eunice Kennedy Shriver National Institute of Child Health and Human Development (NICHD) Fetal Growth Studies standards for Asian populations. The last ultrasound at 28 + 5 weeks gestation confirmed foetal lower limb dysplasia characterised by limb flexion deformity with right clubfoot. The foetal femur, tibial measurements and estimated weight were at 0%, 0% and 3.1% for the gestational age, which were −4.506 SD, −3.402 SD and −1.872 SD below the mean, respectively (Figure 2b). But no abnormalities were detected in the skull, rib cage or other skeletal structures. On the basis of the foetal ultrasound phenotype and the proband′s fracture history, hereditary skeletal system disease was deemed highly probable. Therefore, comprehensive genetic testing was performed on the proband, her husband and foetus, employing methods such as CNV‐seq, trio‐WES and Sanger sequencing.

### 2.2. CNV‐seq

Following the manufacturer′s protocol, genomic DNA was extracted using a NanoWES probe (Berry Genomics, Beijing, China) from peripheral blood samples of the proband (II‐2). The quality of the genomic DNA was subsequently assessed, and 10‐ng genomic DNA was used to generate a PCR‐free fragment library for 6.5 h. With artificial adaptor sequences edited for removal, the raw reads were aligned against the GRCh37 reference genome. Using a smoothness model, the data were analysed based on sequencing read count data and copy number variations (CNVs) were assessed. The reference databases included ClinVar (http://www.ncbi.nlm.nih.gov/clinvar), UCSC Genome Browser (https://genome.ucsc.edu), PubMed (https://pubmed.ncbi.nlm.nih.gov/), Online Mendelian Inheritance in Man (http://www.omim.org/), Database of Genomic Variants (http://dgv.tcag.ca/dgv/app/home), DECIPHER (https://www.deciphergenomics.org/), GeneReviews (http://www.genereviews.org/), ClinGen (https://www.clinicalgenome.org/) and ISCA Database Search (https://www.iscaconsortium.org/). On the basis of the ACMG/ClinGen 2020 technical standards, a document for interpreting sequence variation, the pathogenicity of the candidate CNVs was classified [11].

### 2.3. Trio‐WES

Samples were collected from the proband (II‐2) and her husband (II‐1) via peripheral blood and from the foetus (III‐1) via amniotic fluid. Genomic DNA was extracted using a NanoWES probe (Berry Genomics, Beijing) and sheared into ~200 bp fragments. Afterwards, the DNA fragments were hybrid‐captured with NanoWES (Berry Genomics, China). After qPCR quantification, the 150 bp reads of the libraries were sequenced. The original image file was treated for base calling and generating the original data. Reads were aligned to GRCh38, and PCR duplicates were removed. Variant discovery was performed with the Verita Trekker pipeline (Berry Genomics) supplemented by GATK tools. Afterwards, ANNOVAR and the Enliven Variants Annotation Interpretation System (Berry Genomics) were used to annotate and interpret the variants. Frequency and functional data were extracted from dbSNP (http://www.ncbi.nlm.nih.gov/snp), ClinVar (http://www.ncbi.nlm.nih.gov/clinvar), the UCSC Database (https://genome.ucsc.edu/), gnomAD (http://gnomad.broadinstitute.org/) and OMIM (http://www.omim.org). In silico scores were computed with REVEL, MutationAssessor (http://mutationassessor.org), spliceAI (https://spliceailookup.broadinstitute.org/), MaxEntScan (http://hollywood.mit.edu/burgelab/maxent/Xmaxentscan_scoreseq.html), CADD (http://cadd.gs.washington.edu), FATHMM (http://fathmm.biocompute.org.uk) and RDDC (https://rddc.tsinghua-gd.org/ai/rna-splicer). In accordance with the ACMG/AMP guidelines and SVI general recommendations, the candidate variants were classified into five categories: pathogenic, likely pathogenic, uncertain significance, likely benign and benign [12–14].

### 2.4. RT‐PCR and Sanger Sequencing

RNA was isolated from the proband and reverse‐transcribed into cDNA. The gene‐specific primers used were designed on the basis of the RefSeq transcript sequence (F1: AAGGTTCCCCTGGACGAGAC; F2: CTGCTGGCAAGAGTGGTGAT; R2: AAGCTGAAGTCGAAACCAGC; R1: AGAGTGGCACATCTTGAGGT; F3: CCGTGGTGACAAGGGTGAGA; R3: ACCAGCATCACCAGTGCGA). PCR was conducted as follows: 95°C for 5 min, followed by 35 cycles of 57°C for 30 s and 72°C for 90 s, and extension at 72°C for 7 min. The PCR products were purified by 2% agarose gel electrophoresis. The results of Sanger sequencing were aligned to those of the GRCh38 reference and interpreted with Chromos software.

### 2.5. Minigene Splicing Function Assay

**Figure 3:**
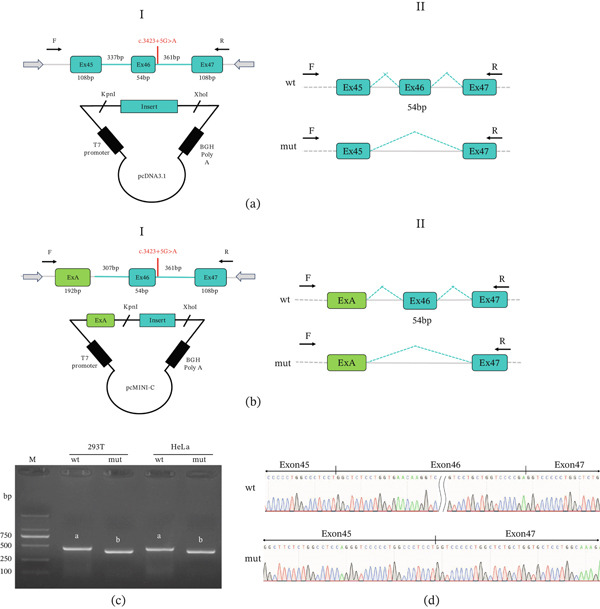
The pcDNA3.1 minigene and pcMINI‐C minigene results of c.3423+5G>A in the *COL1A1* gene (a‐I) pcDNA3.1 vector structure, (a‐II) minigene splicing diagram, (b‐I) pcMINI‐C vector structure, (b‐II) minigene splicing diagram and (c) gene detection via vector agarose gel electrophoresis in HeLa and HEK293T cells, with wild‐type and mutant variants labelled as bands a and b, respectively. The experiment was independently repeated twice with consistent results; representative gels are shown. (d) Results of Sanger sequencing of RT–PCR products.

The impact of candidate variants on *COL1A1* gene splicing was assessed by in vitro minigene experiments. *COL1A1* gene fragments, including Exon 45 (108 bp)–Intron 45 (337 bp)–Exon 46 (54 bp)–Intron 46 (361 bp)–Exon 47 (108 bp), were cloned and inserted into the pcDNA3.1 vector (Figure 3a‐I), whereas different segments, namely, Intron 45 (307 bp)–Exon 46 (54 bp)–Intron 46 (361 bp)–Exon 47 (108 bp), were inserted into the pcMINI‐C vector (Figure 3b‐I). All the wild‐type and mutant pcDNA3.1 and pcMINI‐C constructs were confirmed by Sanger sequencing. Using the conventional restriction and ligation method, each construct was delivered into HeLa and HEK293T cells in separate transfections. After 48 h of incubation, total RNA was isolated and reverse‐transcribed. The cDNA was amplified by PCR. The products were subsequently analysed by 1% agarose gel electrophoresis, after which the wild‐type and mutant sequences, particularly splice variations of the mutant type, were compared.

### 2.6. Review of Splicing Variants in the *COL1A1* Gene

Splicing variants in the *COL1A1* gene were searched in the PubMed database [4, 10, 15–19], including variant location, disease type and a few functional experimental results. In accordance with previous reports [20, 21], these variants were classified into three types, and their associated clinical manifestations were summarised. Moreover, the mechanisms underlying phenotypic variability and the features of C‐terminal variants are briefly discussed.

## 3. Results

### 3.1. Analysis of CNVs

According to CNV‐seq, there were no pathogenic CNVs (seq[hg19](1‐22) × 2,(X■) × 1).

### 3.2. Trio‐WES Identified a Novel Intron Variant in the *COL1A1* Gene

Trio‐WES revealed a novel heterozygous variant in the *COL1A1* gene intron46: NM_000088.4: c.3423+5G>A, which was identified by Sanger sequencing in the proband and her foetus (II‐2 and III‐1) suspected of having OI. The proband′s husband (II‐1) had the wild‐type sequence at this location (Figure 2c). The c.3423+5G>A variant occurs in intron 46, and computational predictions from SpliceAI prediction (score: 0.28) indicated that it may affect splicing (PP3). In ClinVar or HGMD, this has never been reported. Moreover, it was not included in population frequency databases such as the Genome Aggregation Database (gnomAD), the 1000 Genomes Project (1000G), the Human Exome Atlas (ExAC) or the China Genome Database (PM2_Supporting). This variant causes a disease that highly aligns with monogenic bone disorders resulting from *COL1A1* gene variants (PP4). C.3423+5G>A was preliminarily classified as a ‘VUS’ following the ACMG guidelines and SVI General Recommendations (PP3+PM2_Supporting+PP4).

In addition, Trio‐WES identified a heterozygous missense variant in *L1CAM* (NM_000425.5:c.152A>T, p.Asp51Val) in the foetus, inherited from the proband (Figure 2c). This variant has not been reported in population or disease databases, and in silico prediction (REVEL = 0.819) suggested a deleterious effect. However, no *L1CAM*‐related phenotypes were observed in either carrier, and the variant did not segregate with OI severity; therefore, it was classified as a VUS per ACMG guidelines and was not pursued further.

### 3.3. *COL1A1* Gene Splicing Variant Functional Analysis

Consistent results were observed when the pcDNA3.1 and pcMINI‐C vectors were used in the minigene assay. The construction of a minigene plasmid is shown in Figure 3a‐I,b‐I. Minigene splicing assays revealed that c.3423+5G>A can disrupt normal mRNA splicing, and both the pcDNA3.1 and pcMINI‐C vectors confirmed these results (Figure 3a‐II,b‐II). The length of the PCR products differed little between the mutant (b) and wild‐type (a) strains (Figure 3c). Sanger sequencing verified that band b lacks a 54 bp Exon 46 fragment, which confirmed that this variant removes the entire Exon 46, resulting in sequence deletion from nucleotides c.3370 to c.3423 (c.3370_3423del) and amino acid deletion from p.Ser1125 to p.Gly1142 (p.Ser1125_Gly1142del). Exon 46 skipping did not affect the reading frame and caused only an 18‐amino acid (aa) deletion, reducing the protein length from 1464 to 1446 amino acids.

In summary, c.3423+5G>A was reclassified as ‘likely pathogenic (LP)’ on the basis of ACMG guidelines and SVI General Recommendations (PM2_Supporting+PP4+PVS1_Strong); a minigene splicing assay indicated that the variant completely skipped Exon 46 without altering the downstream reading frame, leading to an in‐frame deletion within the protein′s critical functional domain (PVS1_Strong). Supporting evidence from PP4 and PM2_Supporting remains as previously described. Based on the combined genetic and phenotypic assessment, the proband′s subtle blue sclerae and fracture history indicated a diagnosis of mild OI Type IV, whereas the foetus with severe lower limb deformities in utero, including shortened femurs and femoral bowing, was consistent with a diagnosis of OI Type III based on prenatal ultrasound findings. Following comprehensive counselling, we informed the proband and her husband that although the foetus presented with severe lower limb skeletal deformities, some degree of postnatal amelioration could be achieved through surgical intervention. After careful deliberation, the couple ultimately elected to terminate the pregnancy via induction of labour at 29 weeks and 4 days of gestation. The delivered male foetus weighed 1450 g, with a body length of 39 cm and a head circumference of 26 cm. The bilateral tibiae and fibulae were slightly shortened and curved anteriorly, resulting in O‐shaped legs.

### 3.4. Review of Splice Variants of the *COL1A1* Gene

**Figure 4:**
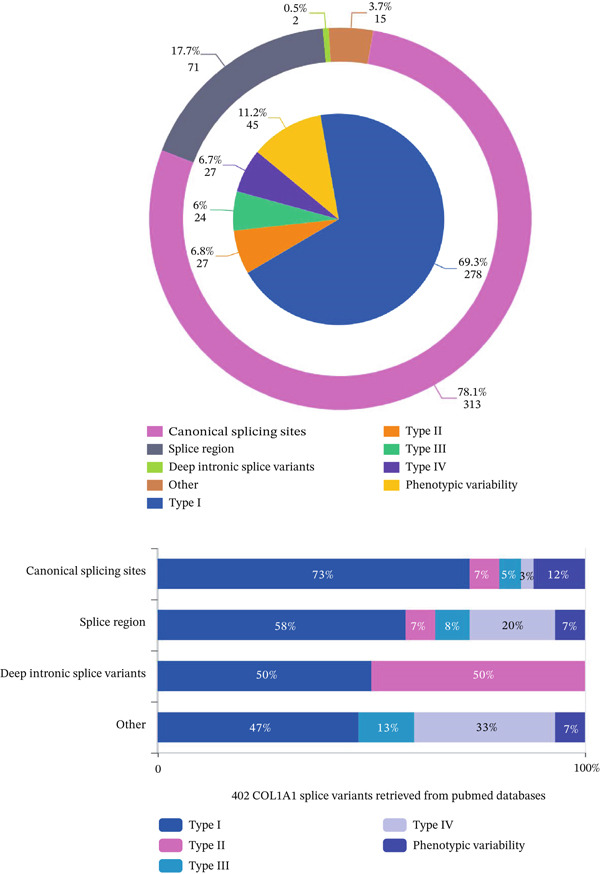
402 *COL1A1* splice variants retrieved from the PubMed database.

According to the literature, *COL1A1* splicing mutations accounted for approximately 22.9% of all *COL1A1* variants identified, with typical splice site variations comprising 14.3% and other intronic variations 8.6% [22]. We retrieved 419 *COL1A1* splice variants from previous literature and reported cases in the PubMed database [4, 10, 15–19], including 15 without phenotypic data and 2 linked to Ehlers–Danlos syndrome (Table S1). In terms of variant location [20, 21], the remaining 402 splice variants were classified into three categories (Figure 4): (1) Variants at canonical splicing sites (77.8%, 313/402) mostly resulted in mild Type I OI (228/313), whereas a minority (21/313) may severely disrupt triple helix formation and represent candidate hotspots for lethal Type II OI [19]. (2) Intron variants in other locations included splice region variants (17.9%, 72/402) and deep intronic splice variants (0.4%, 2/402). Deep intronic splice variants, which primarily lead to large intron retention, may cause significant abnormalities in collagen production and result in more severe phenotypes, such as Type II OI. Splice region variants cause partial exon deletions or small intron retention fragments, affecting collagen quantity. They generally cause nonsevere structural defects and result in relatively mild clinical phenotypes. (3) Other variants (3.7%, 15/402), such as exon variants or fragment loss, are extremely rare and easily overlooked by traditional genetic testing methods, including Sanger sequencing or whole‐exome sequencing. More comprehensive testing approaches are needed [4, 10]. In summary, our review of 402 *COL1A1* splice variants reveals that variant location is broadly associated with clinical severity. The majority of canonical splice site variants (78.1%) result in mild Type I OI, whereas deep intronic splice variants, although rare, are predominantly linked to severe phenotypes, including Type II OI. Splice region variants exhibit a more intermediate and variable phenotype distribution. However, notable phenotypic variability exists even within the same variant category; for instance, 6.2% of canonical splice site variants still resulted in lethal Type II OI, suggesting that additional factors, such as genetic modifiers or allelic heterogeneity, may modulate the clinical outcomes. These findings underscore that while variant location provides a useful framework for prognosis prediction, functional validation remains essential for accurate variant classification, as exemplified by our minigene assay.

## 4. Discussion

OI is a group of genetic disorders primarily characterised by bone fragility and increased fracture susceptibility. Most cases are caused by pathogenic variants in the *COL1A1* and *COL1A2* genes [5]. The phenotypic spectrum of OI extends beyond the skeletal system and typically includes deformities, growth delays and potential involvement of cardiovascular, pulmonary, auditory, dental, and integumentary systems, along with muscle weakness [23]. To date, the underlying pathophysiological mechanisms have not been fully elucidated. The clinical phenotype of OI is highly variable, and variants in the *COL1A1* gene lead mainly to Type I [4], which is consistent with the characteristics of the data we collected (Figure 4 and Table S2).

Minigene assays confirmed that c.3423+5G>A in the C‐terminus (Exons 40–52) of the *COL1A1* gene led to Exon 46 skipping, which is represented as c.3370_3423del p.Ser1125_Gly1142del. This variant was not identified in the gnomAD database, ClinVar database, or domestic or international literature. Only one case recorded in 2009 was retrieved from the LOVD database, suggesting a suspected splicing variant associated with OI Types I and II, with no further information available [24]. In the vicinity of this variant, only c.3423_3423+1del was validated as pathogenic by RNA splicing prediction software. To date, no functional validation experiments for splicing variants in this region have been reported in the literature. C‐propeptide variants in Type I procollagen generally cause severe phenotypes. As shown by Barnes et al. [25, 26], the C‐propeptide drives selective *α*‐chain association and zipper‐like folding from the C‐terminus (Exons 40–52) to the N‐terminus to generate the correct *α*1(I) and *α*2(I) trimers; mutant peptides disrupt this step, delaying assembly and causing ER mislocalisation.

The same *COL1A1* variant demonstrates variable expressivity, resulting in marked phenotypic heterogeneity. Zhytnik et al. reported that 82% of recurrent *COL1A1* and *COL1A2* variants show interfamilial variability, and 32.81% of families present mild‐to‐lethal ranges [27]. A case review revealed that the proband was reclassified as having mild Type IV disease, whereas the foetus had shortened, bowed long bones compatible with Type III disease. This differed from the Type I and II diagnoses listed in the LOVD database. Among the 402 *COL1A1* variants we retrieved, 11.2% (45/402) were associated with documented phenotypic variability (Figure 4). The mechanism remains unclear, and we summarise the putative contributors. First, Pereira et al. demonstrated in utero that an identical *COL1A1* deletion produces variable skeletal phenotypes, whereas mRNA expression and mineral content remain indistinguishable, indicating that variable expressivity is an intrinsic feature of certain *COL1A1* variants [28]. Second, large‐scale genomic analyses have demonstrated that genetic modifiers and ascertainment bias can significantly influence variable expressivity in complex disorders [29]. Specifically, secondary variants, as critical modifiers, may attenuate or exacerbate the clinical severity of the primary *COL1A1* mutation. It is plausible that similar modifying effects operate in *COL1A1*‐related phenotypes, potentially involving interacting genes such as *COL1A2* or other yet unidentified genetic modifiers. In this family, the identified *L1CAM* variant was classified as a VUS, and no *L1CAM*‐related phenotypes were observed in either carrier. Moreover, no literature has reported a pathway association between *L1CAM* and *COL1A1*, nor has any case been documented in which an *L1CAM* variant exacerbates *COL1A1*‐related OI phenotypes. Therefore, this variant is not considered a modifier contributing to the phenotypic variability observed in this pedigree. Moreover, parental somatic/gonadal mosaicism may produce asymptomatic carriers while transmitting a high mutation load to offspring, resulting in severe or even lethal phenotypes [30]. Finally, other factors may include nutritional and other environmental variations between different individuals.

Genetic counselling for *COL1A1*‐related OI should cover both the prognosis and the autosomal dominant inheritance pattern, with particular emphasis on the unpredictability of phenotypic severity. Even in its mildest forms, OI can negatively affect life expectancy [31]. Severe OI cases are primarily at risk of death from respiratory failure secondary to spinal or thoracic deformities [31], whereas mildly affected individuals face increased fracture risk during pregnancy, the postpartum period and breastfeeding [32, 33]. Accordingly, management goals should focus on fracture prevention, pain control and functional independence. Mild Type I patients require routine monitoring, whereas severe Type III/IV patients necessitate multidisciplinary care. Clinically, individuals presenting with recurrent fractures, skeletal deformities, short stature, a family history of OI, or shortened foetal long bones on prenatal ultrasound should raise suspicion of OI and genetic confirmation should be pursued promptly to clarify the prognosis and management. OI caused by *COL1A1* variants follows an autosomal dominant inheritance pattern, conferring a 50% recurrence risk for offspring of an affected parent [5]. More importantly, our study demonstrates considerable intrafamilial variability in OI severity, wherein a mildly affected parent may have offspring carrying identical *COL1A1* variants but exhibiting significantly more severe, even life‐threatening, manifestations [28, 30]. This observation indicates that genotype alone cannot reliably predict phenotype. Therefore, couples should receive thorough counselling regarding the prognosis of OI and be offered reproductive options, including prenatal diagnosis and assisted reproductive technology. Our findings have several clinical implications. With the upgrading of this variant to likely pathogenic, definitive prenatal diagnosis of the foetus can be established, allowing more accurate prognostic counselling for the pregnant woman and her family. This classification also meets the clinical threshold for offering preimplantation genetic testing (PGT), opening up this reproductive option for future pregnancies. Finally, establishing the molecular diagnosis in the proband facilitates the development of personalised long‐term bone health surveillance and fracture prevention strategies.

## 5. Conclusion

In summary, we identified an intron variant in *COL1A1* (NM_000088.4:c.3423+5G>A) as likely pathogenic in the present study. Functional validation demonstrated that this variant causes complete skipping of Exon 46. Our systematic review of 402 *COL1A1* splicing variants further supports a broad association between variant location and clinical severity while also revealing that marked phenotypic variability exists even within the same variant category. This variability underscores that genotype alone is insufficient for accurate prognosis prediction. These findings advance our understanding of *COL1A1* splicing defects and highlight the necessity for further investigation into the molecular determinants driving the variable expressivity of OI.

**Nomenclature**
ACMG: American College of Medical Genetics
CNVs: copy number variations
CNV‐seq: CNV sequencing
*COL1A1*
: collagen Type I Alpha 1 chain
EDS: Ehlers–Danlos syndrome
LOVD: Leiden Open Variation Database
NIPT: non‐invasive prenatal testing
NT: nuchal translucency
OI: osteogenesis imperfecta
OMIM: Online Mendelian Inheritance in Man
WES: whole‐exome sequencing

## Author Contributions

Y.J.Z. and H.B.L. conducted the data analysis and prepared the initial draft. A.L.Y. and S.P.L. reviewed the results and edited the manuscript. W.F.H. coordinated participant recruitment and specimen collection, whereas Y.Z. assembled the figures and tables. B.J. conceived the study design and critically revised the paper. Yujun Zhang and Huibing Liu contributed equally to this work.

## Funding

This study was supported by the Guangdong Province Basic and Applied Basic Research Foundation, No. 2022A1515220087.

## Disclosure

All the authors approved the final manuscript.

## Ethics Statement

This study protocol was approved by the Ethics Board of Nanfang Hospital, Southern Medical University, Guangdong, China (ChiCTR1800016357) and was conducted in accordance with the ethical principles of the Declaration of Helsinki.

## Consent

Consent for publication was obtained from the proband and other family members included in this manuscript.

## Conflicts of Interest

The authors declare that they have no competing interests.

## Supporting Information

Additional supporting information can be found online in the Supporting Information section.

## Supporting information


**Supporting Information 1** Table S1: Supporting information 1.xlsx contains the complete list of 419 COL1A1 splice variants retrieved from the PubMed database.

**Supporting Information 2** Table S2: Supporting information 2.pdf contains the supplementary genetic testing reports of the family.

## Data Availability

The data that support the findings of this study are available on request from the corresponding author. The data are not publicly available because of privacy or ethical restrictions.
