# Supplementary material for: A Novel Splice Variant in the COL1A1 Gene Leads to Exon 46 Skipping and Osteogenesis Imperfecta
Source: Hum Mutat. 2026 Aug 18;2026:2249583. doi: 10.1155/humu/2249583 (PMC13486967; doi:10.1155/humu/2249583)
Supplement: Supplementary file 1 [file HUMU-2026-2249583-s001.pdf]

Supplementary Data

Report of Genetic Analysis by CNV-seq and Trio-based Whole Exome Sequencing

1. CNV-seq Findings

According to the International System for Human Cytogenomic Nomenclature (ISCN 2016):

Karyotype: seq[hg19] (1-22)×2, (X■)×1

Interpretation: No pathogenic chromosomal aneuploidies or known disease-associated CNVs >100 kb were detected within the resolution of the assay.

Figure S1: CNV-seq profile

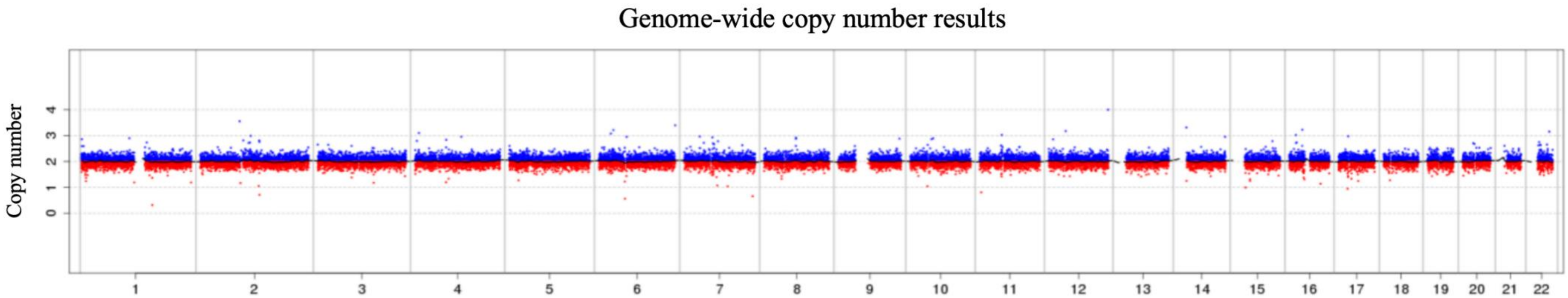

2. Trio-WES Findings

Gene	Inheritance	Genomic Position (hg38)	HGVS	Variant Type	Genotype			ACMG Classification	Evidence	Associated Disorders/Phenotypes
					Fetus	Proband	Husband			
COL1A1	AD	chr17:50187479-50187479 intron46	NM_000088.4: c.3423+5 G>A	Intron variant	het	het	wild	Likely pathogenic	PVS1_Strong+PP4 +PM2_Supporting	Osteogenesis imperfecta, Caffey disease, Ehlers-Danlos syndrome type 1
L1CAM	XLR	chrX:153872637-153872637 exon3	NM_000425.5: c.152A>T (p.D51V)	Missense variant	hemi	het	wild	VUS	PP3_Moderate+PM2_Supporting	Partial agenesis of corpus callosum, Congenital hydrocephalus, MASA syndrome

Table S1: Summary of COL1A1 and L1CAM variants. ACMG: American College of Medical Genetics and Genomics; VUS: Variant of Uncertain Significance; AD: Autosomal Dominant; XLR: X-Linked Recessive; het: heterozygous; hemi: hemizygous; HGVS: Human Genome Variation Society; hg38: Human Genome 38.

Figure S2: Sequencing chromatograms for COL1A1 and L1CAM

COL1A1:NM_000088.4:c.3423+5G>A

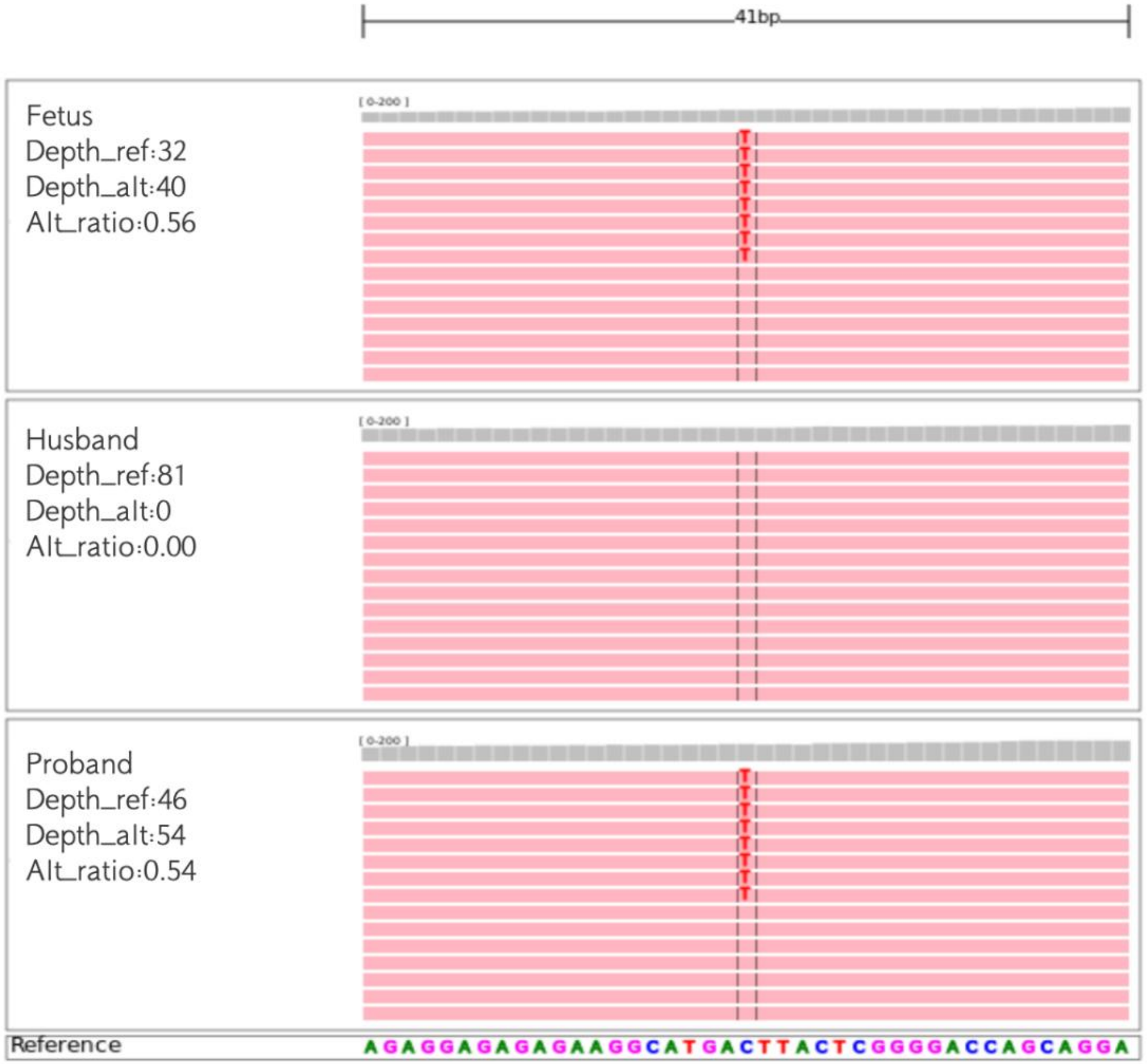

L1CAM:NM_000425.5:c.152A>T(p.D51V)

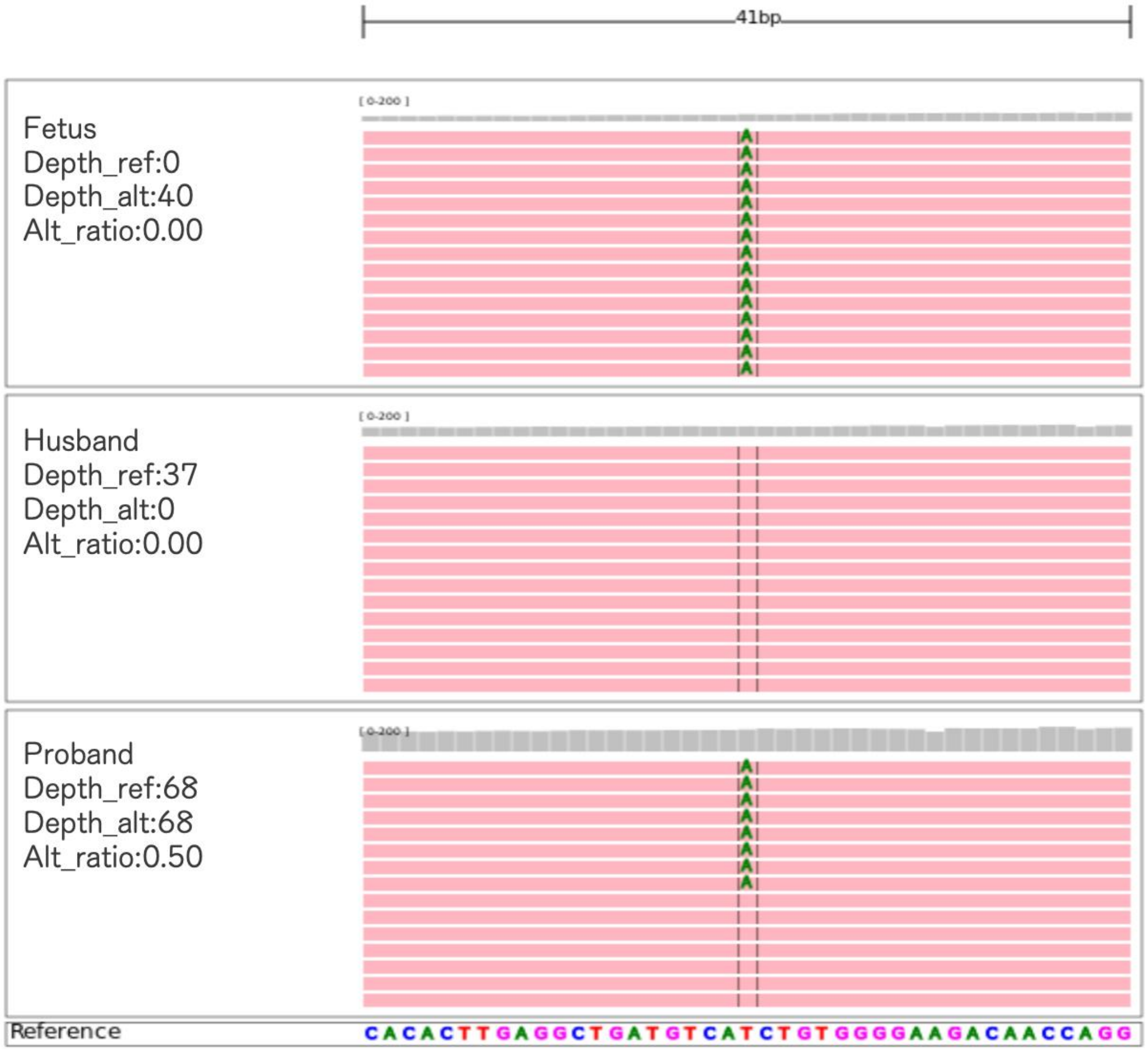

Table S2: Coverage statistics for exome sequencing.

Sample	Sequencing Method	20× Coverage (%)
Fetus	Whole Exome	98.78%
Proband	Whole Exome	99.53%
Husband	Whole Exome	99.53%

References

1.Richards S, Aziz N, Bale S, et al. Standards and Guidelines for the Interpretation of Sequence Variants: A Joint Consensus Recommendation of the American College of Medical Genetics and Genomics and the Association for Molecular Pathology[J]. Genetics in Medicine Official Journal of the American College of Medical Genetics, 2015, 17(5):405.

2.Kalia S S, Adelman K, Bale S J, et al. Recommendations for reporting of secondary findings in clinical exome and genome sequencing, 2016 update (ACMG SF v2.0): a policy statement of the American College of Medical Genetics and Genomics[J]. Genetics in Medicine Official Journal of the American College of Medical Genetics, 2017, 19(2):249.

3.Kearney H M, Thorland E C, Brown K K, et al. American College of Medical Genetics standards and guidelines for interpretation and reporting of postnatal

constitutional copy number variants[J]. Genetics in Medicine Official Journal of the American College of Medical Genetics, 2011, 13(7):680-5.

4.Ahmad N Abou Tayoun, et al. Recommendations for interpreting the loss of function PVS1 ACMG/AMP variant criterion[J]. Human Mutation, 2018.

5. Biesecker L G , Harrison S M . The ACMG/AMP reputable source criteria for the interpretation of sequence variants[J]. Genetics in Medicine, 2018, 20(12).

6.Rajarshi, Ghosh, Steven, et al. Updated recommendation for the benign stand-alone ACMG/AMP criterion.[J]. Human mutation, 2018
